# Determinants and gaps influencing zero-dose immunization and maternal Health service utilization in Nigeria: a cross-sectional household survey across six Nigerian states

**DOI:** 10.1080/16549716.2026.2660488

**Published:** 2026-04-24

**Authors:** Adewale Akinjeji, Olufunke Fasawe, Abubakar Abba, Ayo Stephen Adebowale, Helena Nordenstedt, William Reidy, Olalekan A. Uthman, Remi Oladigbolu, Jaran Eriksen

**Affiliations:** a Department of Global Public Health, Karolinska Institutet, Stockholm, Sweden; b Clinton Health Access Initiative, Abuja, Nigeria; c Department of Epidemiology and Medical Statistics, Faculty of Public Health, College of Medicine, University of Ibadan, Ibadan, Nigeria; d Department of Medical Specialties, Danderyd University Hospital, Stockholm, Sweden; e Department of Epidemiology, ICAP Columbia University, New York, USA; f Warwick Applied Health, Warwick Medical School, University of Warwick, Coventry, UK; g ICAP Global Health, Abuja, Nigeria; h Department of Clinical Sciences and Education, Karolinska Institutet, Stockholm, Sweden; i Unit of Infectious Diseases/Venhälsan, Stockholm, South General Hospital, Stockholm, Sweden

**Keywords:** Maternal health, child immunization, zero-dose children, RMNCH, Nigeria

## Abstract

**Background:**

Nigeria continues to experience high maternal and child mortality, despite extensive health reforms and international investment. Missed opportunities across the continuum of care, antenatal care (ANC), skilled birth attendance, facility-based delivery, and child immunization, contribute to these outcomes. Children who receive no vaccines (zero-dose) reflect a key vulnerability in service delivery.

**Objective:**

To identify factors associated with zero-dose immunization and patterns of maternal healthcare utilization among women in six Nigerian states.

**Methods:**

A cross-sectional household survey was conducted in Gombe, Kaduna, Kano, Katsina, Lagos, and Niger states. A total of 1,958 women aged 15–49 years with recent live births were interviewed. Structured questionnaires captured data on ANC attendance, delivery location and attendant, immunization status, and socio-demographic characteristics. Descriptive statistics, chi-square tests, and multinomial logistic regression were used to assess predictors of maternal and child health service utilization.

**Results:**

Only 31% of women received complete care; 58% received partial care, and 11% received none. Zero-dose prevalence was significantly higher among children whose mothers had fewer than four ANC visits (35%), delivered at home (35%), or were attended by unskilled providers (39%). Lower zero-dose rates were found among those delivered in facilities (16%) or by skilled attendants (19%). Key predictors included maternal and paternal education, socioeconomic status, geographic location (notably Kano and Katsina), and maternal age.

**Conclusions:**

Significant disparities in maternal and child health service utilization remain. Expanding ANC coverage, increasing skilled facility-based deliveries, and ensuring postnatal immunization linkage are essential to reduce zero-dose prevalence and promote equity in low-resource settings.

## Paper Context

- **Main findings:** Women who received fewer than four antenatal care visits, delivered at home, or used unskilled birth attendants were significantly more likely to have children who received no routine vaccinations.
- **Added knowledge:** This study uniquely links maternal healthcare utilization across the continuum of care with zero-dose immunization outcomes, highlighting compounded service gaps in low-resource settings.
- **Global health impact for policy and action:** Targeted, equity-focused interventions that integrate maternal health services with immunization efforts are essential to reducing zero-dose prevalence and achieving universal health coverage in underserved populations.

## Background

Nigeria, the most populous country in Africa, continues to experience complex and systemic challenges in reproductive, maternal, newborn, and child health (RMNCH), despite decades of national health reforms and substantial international investment [1]. Health outcomes for women and children remain among the poorest globally [2]. According to the World Health Organization (WHO), Nigeria accounted for more than 25% of global maternal deaths, with a maternal mortality ratio (MMR) of almost 1000 per 100,000 live births in 2023 [3]. Neonatal mortality is also alarmingly high, with 60 deaths per 1,000 live births [4]. Also, recent estimates by WHO and UNICEF (2023) further highlight Nigeria’s significant burden of zero-dose and under-immunized children, with over 2.1 million not receiving any routine vaccines as of 2023 [5,6]. This highlights significant service delivery gaps and inequities in access to essential health interventions across the RMNCH continuum of care, reflecting persistent shortcomings in the quality, continuity, and accessibility of maternal and newborn care services.

Evidence consistently affirms the importance of quality antenatal care (ANC), institutional delivery, skilled birth attendance (SBA), and routine immunization in reducing preventable maternal and child deaths [7]. WHO recommends a life-course and continuum-of-care approach that ensures integrated and quality services at each stage, from pregnancy and childbirth to childhood immunizations [8]. Skilled care during pregnancy and childbirth mitigates the risks of obstetric complications and stillbirth [9,10], while immunization remains one of the most cost-effective public health interventions, preventing an estimated 2.5 million child deaths annually [11]. Roughly one in five under-five deaths are attributable to vaccine-preventable diseases, emphasizing the critical role of immunization in child survival strategies [12].

Over the years, Nigeria has implemented a range of policies and programs to address these persistent challenges, particularly by strengthening service delivery at the primary health care (PHC) level. These include the revitalization of PHC under the National Primary Health Care Development Agency (NPHCDA), the Midwives Service Scheme to improve skilled birth attendance, the introduction of the Basic Health Care Provision Fund (BHCPF) to enhance equitable financing, and the Nigeria Strategy for Immunization and Primary Health Care System Strengthening [13,14]. Other initiatives, such as the National Reproductive Health Policy and the Integrated Maternal, Newborn, and Child Health Strategy, have aimed to promote quality and continuity of care throughout the RMNCH continuum [15]. Despite these efforts, significant gaps remain, as highlighted by findings from the recent National Health and Demographic Survey (NHDS).

The 2024 Nigeria Demographic and Health Survey (NDHS) shows only 52% of women attended at least 4 ANC visits [16], and just 46% of deliveries were assisted by skilled providers, with pronounced rural-urban disparities. In many rural areas, traditional birth attendants (TBAs) remain the primary providers despite lacking formal clinical training [17]. Childhood immunization coverage is similarly suboptimal; only 36% of children aged 12–23 months are fully vaccinated according to the 2022 Multiple Indicator Cluster Survey (MICS), with wide regional and socioeconomic inequalities [18]. Barriers include misinformation, vaccine hesitancy, supply chain weaknesses, and entrenched socio-cultural norms [19,20].

These interconnected challenges of low ANC utilization, home delivery, unskilled birth attendance, and zero-dose immunization represent missed opportunities that collectively amplify risks of preventable morbidity and mortality. This study contributes to the evidence base by examining these four outcomes not as isolated events, but as interrelated components of the care continuum. While previous research has often examined single RMNCH outcomes, this study adopts a holistic lens to identify shared and distinct determinants of sub-optimal ANC, home delivery, unskilled birth attendance, and zero-dose immunization in Nigeria, with the aim to inform targeted and integrated health policy and interventions. The aim of this study is to examine factors associated with two key outcomes: (1) zero-dose immunization status among children and (2) a composite care continuum score reflecting the four essential RMNCH indicators mentioned above, to inform targeted and integrated health policy and interventions.

## Methods

### Study design

#### Household survey Methodology

This study is a component of a broader research study conducted by the Clinton Health Access Initiative (CHAI) Nigeria to evaluate the effect of Collaborative Learning project on data use for action to improve primary health care (PHC) facilities performance and equitable immunization coverage in Nigeria. Our study employed a cross-sectional household survey to assess key indicators related to maternal and child health, including immunization coverage, ANC, place of delivery, and skilled birth attendance. The survey was conducted from January 21^st^ to 24 March 2024 across six Nigerian states (Lagos, Gombe, Niger, Kaduna, Kano, and Katsina) with a focus on communities surrounding BHCPF supported health facilities. The BHCPF is a government-backed financing mechanism designed to ensure at least one fully functional primary healthcare facility per ward (administrative division within a local government area) by supporting public and private PHC facilities with essential services, infrastructure, and workforce funding.

## Survey

This cross-sectional household survey was conducted as a stand-alone baseline assessment designed specifically to evaluate maternal and child health service utilization and zero-dose immunization prior to the initiation of the Collaborative Learning project. Although implemented alongside broader programmatic monitoring activities in the study states, the questionnaire, sampling strategy, and analytic framework were designed specifically for this survey.

## Study population

The six Nigerian states included in our study were strategically selected to represent the country’s geographic, socio-cultural, and health system diversity. This purposeful selection ensured inclusion of both urban and rural populations and allowed for comparative analysis across states. The target population was women of reproductive age (15–49 years) who had experienced at least one live birth within the past 59 months preceding the survey, as well as children aged 6–59 months residing in the same households. This population group was selected to enable the assessment of key maternal and child health service utilization indicators, including ANC attendance, delivery practices, and childhood immunization status. Only women who provided signed, informed consent were interviewed. The consent process included reading the consent statement aloud to the participant to ensure comprehension, after which participants appended their signatures or thumbprints to the consent section of the data collection form. Data were collected at the household level using structured questionnaires. All women in each household were first listed, and one eligible woman was randomly selected for interview. If the selected woman declined participation, the selection process was repeated. Women residing in the same building but belonging to different households (i.e. with different husbands or household heads) were treated as separate households. Women outside the 15–49 year age range, those without a live birth in the 59 months preceding the survey, households without an eligible child aged 6–59 months, temporary residents, and individuals who declined to provide informed consent or were unable to participate at the time of the interview were excluded from the survey.

## Data collection procedures

The household survey sample size was calculated using an estimated 43.4% prevalence of children not fully immunized with the third dose of Pentavalent vaccine (Penta3) [21]. This estimate incorporated a design effect of 1.5, a 5% precision level, and a 10% expected non-response rate. The assumed prevalence of 43.4% was drawn from available national and subnational immunization estimates at the time of study design and was used solely for sample size calculation. Although zero-dose status was the primary outcome, Penta3 coverage was used for sample size estimation because it is a routinely reported and reliable indicator in immunization data and provides a conservative estimate of service gaps; Penta1 coverage was not consistently reported nationally.

Based on these assumptions, a sample size of 3,775 women across the six Nigerian states was initially planned, but due to budget constraints, the final sample size was reduced to 1,958. These reductions were applied proportionally across all study states and sampling strata to preserve geographic representation and the overall study design.

**Figure 1.**
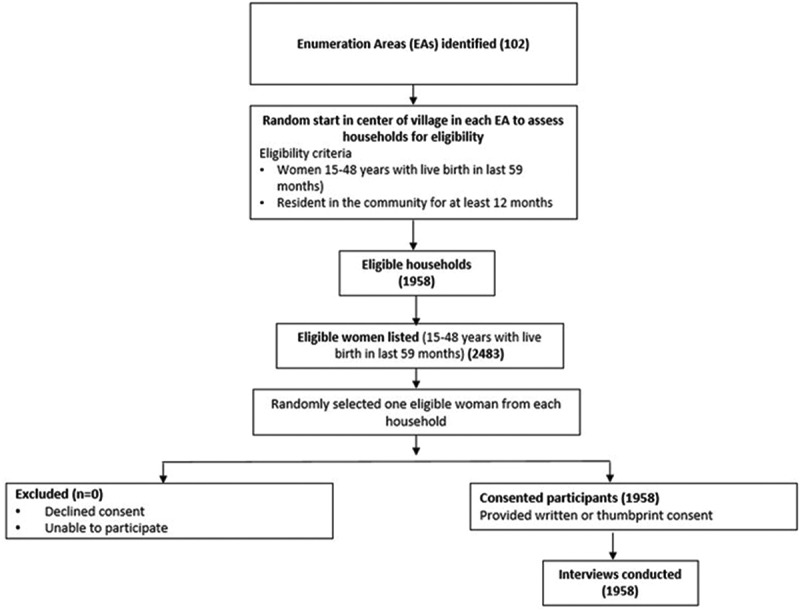
Flow diagram of participant selection and inclusion in the cross-sectional household.

The data-collection followed a multi-stage probability cluster sampling approach, ensuring representativeness and robustness in estimating key indicators. Enumeration areas (EAs) were randomly selected proportional to the estimated survey population size from within the catchment areas of BHCPF-supported health facilities. A multistage sampling technique was used. In Stage 1, six states were purposively selected based on project priorities and a high burden of zero-dose children. In Stage 2, enumeration areas within each selected state were listed and randomly selected for the survey. In Stage 3, households with at least one child under five years of age within each enumeration area were listed, and 16 households were systematically selected for interview. This resulted in a total sample of 1,958 households across the six states (Figure 1). For each selected household, all children under five were listed, and their mothers or primary caregivers were interviewed.

Interviews were conducted with mothers or primary caregivers, and data were collected for all eligible children. Data were collected using a structured questionnaire that was adapted from the Nigeria Demographic and Health Survey (NDHS), a nationally validated instrument for population-based health research in Nigeria. The NDHS questionnaire was shortened and included only questions that were relevant to the research while retaining standard NDHS definitions and indicators. The questionnaire, which covered ANC, delivery location, type of birth attendant, immunization status, and key socio-economic and demographic factors. The survey utilized Computer-Assisted Personal Interviewing, a process that involves administering surveys using electronic tablet devices to record responses directly in a digital system, to ensure accurate and efficient data entry. Enumerators were trained fieldworkers fluent in local languages, and received intensive instruction during pre-field work training sessions on survey tools, ethics, consent, and digital data collection. The questionnaire was pre-tested among 52 eligible women we aimed for around 50 women) in non-study areas to assess clarity, flow, and cultural appropriateness. Feedback from interviewers was used to refine question wording, response options, and overall structure prior to final data collection. Quality assurance was maintained through supervision by trained coordinators, daily data reviews, and real-time monitoring using electronic dashboards throughout the fieldwork period.

## Independent variables

Independent variables consisted of maternal and child healthcare service utilization and socio-demographic, economic factors and household characteristics.

### Maternal and child healthcare service utilization

This included ANC attendance (attended at least one ANC visit during pregnancy), place of delivery (home vs. health facility), and type of birth attendant (skilled vs. unskilled), and zero-dose immunization status (not received even a single vaccine dose). Skilled birth attendance are deliveries assisted by a doctor, nurse, midwife, or auxiliary nurse/midwife, while deliveries assisted by traditional birth attendants, relatives, friends, or with no attendant are classified as unskilled.

### Socio-demographic and economic factors

These included maternal age (categorized: <20, 20–34, 35–49), education (primary and below vs. secondary and above), ethnicity (Hausa, Igbo, Yoruba, Others), religion (Christianity or Islam), marital status (married/cohabiting, never married, divorced, separated, widowed), and employment status (employed vs. unemployed).

### Household SES

Socioeconomic status was assessed using a study-specific composite index developed by the research team, drawing on approaches commonly used in demographic and health surveys. The index was constructed using household asset ownership, housing characteristics, and access to basic services. Each indicator was assigned a score of one, and scores were summed to generate a total ranging from 0 to 11. Based on the distribution of scores, households were categorized into low (0–3), middle (4–7), and high (8–11) socioeconomic status.

### Paternal characteristics

Father’s education and employment status were also included, categorized in the same way as the maternal education and employment status indicators.

### Child-specific factors

These included birth order and gender.

These variables were selected to reflect key social and economic determinants influencing access to and use of maternal and child health services in Nigeria. In households with more than one eligible child, data were collected for a single index child to minimize intra-household clustering. The youngest eligible child was selected using a predefined rule, and all vaccination and care indicators were assessed for that child only.

## Dependent variables

### Outcome variable: zero-dose immunization status

The outcome variable ‘Zero-Dose status’, was defined using the WHO definition of zero-dose [22]: children aged 6–59 months who had not received any routine vaccinations were classified as zero-dose, while those with at least one dose classified as non-zero-dose [22].

### Outcome variable: care utilization pattern

The outcome variable ‘Care Utilization Pattern’ was developed as a multinomial categorical measure capturing distinct levels of maternal and child health service engagement. This composite variable was derived from four evidence-based service indicators that represent critical touchpoints along the maternal-child health continuum: (1) adequate antenatal care attendance (≥4 ANC visits vs. <4 visits), (2) place of delivery (health facility vs. home), (3) type of birth attendant (skilled vs. unskilled), and (4) child vaccination status (zero dose vs. non-zero-dose).

Each woman received a composite score ranging from 0 to 4 based on the number of recommended services received. This score was then categorized into three mutually exclusive care utilization patterns:

- ‘Complete Care’ (reference category): Receipt of all four recommended services (score = 4), representing optimal engagement with the formal health system and adherence to WHO-recommended standards for comprehensive maternal and child health care
- ‘No Care’: Absence of all four services (score = 0), indicating complete reliance on informal or traditional care systems without engagement with formal healthcare services
- ‘Partial Care’: Receipt of one to three of the four recommended services (score = 1–3), representing fragmented or suboptimal service utilization where women engage with some but not all components of the maternal-child health continuum

This three-category classification aligns with the continuum of care framework [23] and provides actionable insights for interventions targeting different levels of health system engagement.

## Statistical analysis

A bivariate descriptive analysis was conducted to explore how socioeconomic status, education, religion, birth characteristics, maternal and paternal characteristics and state-level differences may influence maternal service utilization and the likelihood of children receiving zero or at least one dose of routine childhood vaccines.

Chi-square analysis was conducted to identify predictors of zero-dose immunization status. Explanatory variables included ANC attendance, place of delivery, birth attendant type, and sociodemographic characteristics. Statistical significance was determined at an alpha level of 0.05, and findings were interpreted with consideration of their health system and equity implications.

For the care utilization pattern outcome variable, a separate analysis was conducted, comprising both descriptive and inferential statistical methods to explore factors associated with maternal and child health service utilization and child immunization outcomes along the continuum of care. Descriptive statistics were used to summarize the distribution of key independent variables. Chi-square tests were employed to assess bivariate associations and determine statistical significance at the level of Care Category.

For multivariable analysis, multinomial logistic regression was used to model the three-level Care Category outcome. Complete care served as the base outcome. Covariates included in the model were selected based on theoretical relevance and significance in bivariate analysis (*p* < 0.05). Adjusted odds ratios (AORs) and 95% confidence intervals (CIs) were reported. All analyses were conducted using the Statistical Package for the Social Sciences (SPSS) version 25 [24].

## Model fit and specifications

We evaluated multicollinearity using variance inflation factors (VIF) and tolerance statistics, examined the presence of influential outliers, and calculated the adjusted R-squared to assess overall model fit [25]. Multicollinearity was assessed by examining VIF values for all predictor variables. Following established guidelines, VIF values greater than 10 or a mean VIF exceeding 6 would indicate problematic multicollinearity requiring remediation. Tolerance values below 0.1 would similarly suggest concerning levels of multicollinearity among predictors. Particular attention was given to conceptually related variables – such as religion, ethnic group, and state, given their potential for overlap in the study context.

## Results

This section presents our findings on maternal and child healthcare service utilization where we aimed to determined factors associated with zero-dose immunization status among children and develop a composite score for accessing the continuum of care.

## Descriptive statistics

A total of 1,958 women of reproductive age participated in the survey across six Nigerian states.

Table 1 presents the distribution of key maternal and child health indicators – ANC visits, place of delivery, birth attendant type, and zero-dose immunization status – across selected sociodemographic and contextual variables. About 20% of women had fewer than four ANC visits. Notably, suboptimal ANC attendance was highest in Kano (38%) and Katsina (34%), and lowest in Lagos (12%) and Kaduna (14%). Poor ANC uptake was more common among women with lower education levels, younger maternal age (<20 years), low SES, and those from Yoruba ethnic backgrounds or without formal employment.

**Table 1.** Overview of maternal health service utilization and zero-dose immunization status in six states in Nigeria.

	ANC Visits		Place of delivery (Home or Health Facility)		Birth Attendant Type (Skilled or Unskilled)		Immunization status in children ≤ 5 years	
	Below < 4 visits	ANC +4 visits	Home	Health Facility	Unskilled Birth Attendant (TBA/Unattended delivery)	Skilled Birth Attendant	Zero- Dose	At least one dose
	N (%)	N (%)	N (%)	N (%)	N (%)	N (%)	N (%)	N (%)
	334 (20.3)	1308 (79.7)	1061 (54.2)	897 (45.8)	777 (39.7)	1181 (60.3)	487 (26.8)	1331 (73.2)
**State**								
Gombe	34 (23.9)	108 (76.1)	51 (33.6)	101 (66.4)	36 (23.7)	116 (76.3)	25 (17.2)	120 (82.8)
Kaduna	26 (14.0)	160 (86.0)	161 (59.6)	109 (40.4)	92 (34.1)	178 (65.9)	38 (15.6)	205 (84.4)
Kano	119 (37.5)	198 (62.5)	296 (77.9)	84 (22.1)	263 (69.2)	117 (30.8)	121 (32.8)	248 (67.2)
Katsina	90 (33.5)	179 (66.5)	352 (69.0)	158 (31.0)	288 (56.5)	222 (43.5)	222 (49.0)	231(51.0)
Lagos	10 (12.0)	73 (88.0)	51 (16.7)	255 (83.3)	23 (7.5)	283 (92.5)	29 (10.6)	244 (89.4)
Niger	55 (19.2)	231 (80.8)	150 (44.1)	190 (55.9)	75 (22.1)	265 (77.9)	52 (15.5)	283 (84.5)
**Maternal Age**								
<20	22 (34.4)	42 (65.6)	72 (63.7)	41 (36.3)	51 (45.1)	62 (54.9)	39 (39.4)	60 (60.6)
20–34	240 (26.5)	666 (73.5)	715 (52.8)	640 (47.2)	510 (37.6)	845 (62.4)	336 (26.6)	926 (73.4)
35–49	72 (23.0)	241 (77.0)	274 (55.9)	216 (44.1)	216 (44.1)	274 (55.9)	112 (24.5)	345 (75.5)
**Maternal Education**								
Primary and below	134 (30.3)	308 (69.7)	511 (76.4)	158 (23.6)	410 (61.3)	259 (38.7)	234 (37.7)	386 (62.3)
Secondary and above	104 (21.7)	376 (78.3)	275 (37.1)	466 (62.9)	171 (23.1)	570 (76.9)	127 (18.5)	561 (81.5)
**Ethnic Group**								
Hausa	194 (19.4)	806 (80.6)	570 (50.7)	586 (49.3)	395 (31.2)	761 (65.8)	255 (23.5)	829 (76.5)
Igbo	7 (18.4)	31 (81.6)	45 (78.9)	12 (21.1)	39 (68.4)	18 (31.6)	19 (38.8)	30 (61.2)
Yoruba	40 (29.4)	96 (70.6)	159 (80.3)	39 (19.7)	131 (66.2)	67 (33.8)	83 (45.4)	100 (54.6)
Others	93 (19.9)	375 (80.1)	287 (52.5)	260 (47.5)	212 (38.8)	335 (61.2)	130 (25.9)	372 (74.1)
**Religion**								
Christianity	18 (14.2)	109 (85.8)	55 (18.2)	248 (81.8)	21 (6.9)	282 (93.1)	29 (10.8)	240 (89.2)
Islam	315 (27.3)	840 (72.7)	1005 (60.8)	649 (39.2)	755 (45.6)	899 (54.4)	458 (29.6)	1090 (70.4)
**Marital Status**								
Divorced	5 (33.3)	10 (66.7)	11 (50.0)	11 (50.0)	4 (40.0)	6 (60.0)	4 (21.1)	15 (78.9)
Married/Cohabiting	268 (24.9)	807 (75.1)	880 (53.9)	754 (46.1)	10 (58.8)	7 (41.2)	393 (25.8)	1132 (74.2)
Separated	2 (33.3)	4 (66.7)	4 (40.0)	6 (60.0)	129 (46.9)	146 (53.1)	3 (37.5)	5 (62.5)
Never Married	56 (31.6)	121 (68.4)	154 (56.0)	121 (44.0)	626 (38.3)	1008 (61.7)	84 (33.3)	168 (66.7)
Widowed	3 (30.0)	7 (70.0)	12 (70.6)	5 (29.4)	8 (36.4)	14 (63.6)	3 (21.4)	11 (78.6)
**Socioeconomic Status**								
Low LS (0–3)	232 (29.9)	545 (70.1)	787 (68.1)	369 (31.9)	612 (52.9)	544 (47.1)	357 (33.5)	709 (66.5)
Middle LS (4–7)	94 (20.5)	365 (79.5)	260 (36.7)	449 (63.3)	157 (22.1)	552 (77.9)	118 (17.8)	546 (82.2)
High LS (8–11)	8 (17.0)	39 (83.0)	14 (15.1)	79 (84.9)	8 (8.6)	85 (91.4)	12 (13.6)	76 (86.4)
**Maternal Employment**								
Yes	197 (23.0)	659 (77.0)	669 (52.9)	595 (47.1)	499 (39.5)	765 (60.5)	265 (22.5)	914 (77.5)
No	137 (32.1)	290 (67.9)	392 (56.5)	302 (43.5)	278 (40.1)	416 (59.9)	222 (34.7)	417 (65.3)
**Paternal Education**								
Primary and below	91 (27.7)	237 (72.3)	394 (75.0)	131 (25.0)	322 (61.3)	203 (38.7)	182 (37.8)	300 (62.2)
Secondary and above	177 (23.7)	570 (76.3)	486 (43.8)	623 (56.2)	304 (27.4)	805 (72.6)	211 (20.2)	832 (79.8)
**Paternal Employment**								
Yes	218 (24.2)	684 (75.8)	690 (51.3)	655 (48.7)	498 (37.0)	847 (63.0)	285 (22.6)	976 (77.4)
No	50 (28.9)	123 (71.1)	190 (65.7)	99 (34.3)	128 (44.3)	161 (55.7)	108 (40.9)	156 (59.1)
	N (%)	N (%)	N (%)	N (%)	N (%)	N (%)	N (%)	N (%)
	334 (20.3)	1308 (79.7)	1061 (54.2)	897 (45.8)	777 (39.7)	1181 (60.3)	487 (26.8)	1331 (73.2)
**Birth Order**								
1st	253 (22.4)	877 (77.6)	889 (64.7)	486 (35.3)	659 (47.9)	716 (52.1)	368 (28.7)	914 (71.3)
2nd	59 (17.0)	289 (83.0)	138 (34.5)	262 (65.5)	93 (23.2)	307 (76.8)	85 (23.0)	284 (77.0)
3rd	22 (13.4)	142 (86.6)	34 (18.6)	149 (81.4)	25 (13.7)	158 (86.3)	34 (20.4)	113 (79.6)
**Child Gender**								
Female	156 (19.6)	639 (80.4)	514 (53.8)	441 (46.2)	377 (39.5)	578 (60.5)	224 (25.2)	664 (74.8)
Male	178 (21.0)	669 (79.0)	547 (54.5)	456 (45.5)	400 (39.9)	603 (60.1)	263 (28.3)	667 (71.7)

ANC = Antenatal care; LS = Living Standard. **Note**: The total numbers in each category may not add up due to missing or incomplete responses for some variables.

Regarding place of delivery, 46% of births occurred in health facilities. Facility-based deliveries were lowest in Kano (22%) and Katsina (31%), but highest in Lagos (83%) and among ethnic groups categorized as ‘Hausa’ (49%). Facility delivery was positively associated with higher maternal education and higher SES.

Use of a skilled birth attendant was reported in 60% of cases. Unskilled delivery was most prevalent in Kano (69%) and Katsina (57%), and least common in Lagos (8%) and among the Hausa ethnic group (31%). Higher use of skilled attendants was correlated with higher education, higher SES, and urban residence. For child immunization, 27% of children were classified as zero-dose, meaning they had not received any routine vaccinations. The highest zero-dose prevalence was observed in Katsina (49%), Kano (33%), among the Yoruba ethnic group (45%), and among households with low SES (34%).

## Associations between maternal health service utilization and zero-dose immunization status

Table 2 presents the associations between ANC attendance, place of delivery, birth attendant type and zero-dose immunization status, with zero-dose defined as the absence of any routine vaccination in children aged 6–59 months. Our analysis shows statistically significant associations between maternal health service utilization and zero-dose immunization status among children under five. Children whose mothers attended fewer than 4 ANC visits had a higher proportion of zero-dose status (35.0%) compared to those whose mothers attended 4 or more visits (17.1%) (*p* = 0.017).

**Table 2.** Associations between of ANC, place of delivery, birth Attendant types and zero-dose immunization status.

	Immunization status in children ≤ 5 years		
	Zero dose	At least one dose	P-Value
**ANC**			
Below 4 ANC Visits	112 (35.0)	208 (65.0)	0.017
4 ANC visits and above	156 (17.1)	758 (82.9)	
**Home or Facility Delivery**			
Home	347 (35.4)	634 (64.6)	<0.001
Health Facility	140 (16.7)	697 (83.3)	
**Skilled Birth Attendant**			
Un-Skilled Birth Attendant (TBA/Unattended)	278 (38.8)	438 (61.2)	<0.001
Skilled Birth Attendant	209 (19.0)	893 (81.0)	

ANC = Antenatal care, TBA = Traditional Birth Attendant.

Home deliveries were associated with a significantly higher proportion of zero-dose children (35.4%) than facility-based deliveries (16.7%) (*p* < 0.001). Children delivered by unskilled attendants had the highest proportion of zero-dose status (38.8%) compared to those delivered by skilled birth attendants (19.0%) (*p* < 0.001).

Table 3 presents the distribution of maternal and child healthcare service utilization along the continuum of care across key sociodemographic and household characteristics. Overall, 11% of women received no care, 58% received partial care, and 31% received complete care. Significant variation was observed across states, maternal education, socioeconomic status, ethnicity, and religion (*p* < 0.001 for most variables). Geographical disparities were pronounced. Katsina and Kano recorded the highest proportions of women with no care (24% and 15%, respectively), while Lagos and Gombe had the highest proportions receiving complete care (65% and 41%).

**Table 3.** Bivariate analysis of care utilization patterns by maternal, household, and child characteristics.

	No care	Partial Care	Complete Care	Total	P-value
	N (%)	N (%)	N (%)	N (%)	
	217 (11.1)	1140 (58.2)	601 (30.7)	1958 (100)	
**State**					
Gombe	11 (7.2)	68 (44.7)	73 (48.0)	152 (100)	
Kaduna	14 (5.2)	173 (64.1)	83 (30.7)	270 (100)	
Kano	55 (14.5)	276 (72.6)	49 (12.9)	380 (100)	
Katsina	122 (23.9)	328 (64.3)	60 (11.8)	510 (100)	
Lagos	4 (1.3)	104 (34.0)	198 (64.7)	306 (100)	
Niger	11 (3.2)	191 (56.2)	138 (40.6)	340 (100)	<0.001
**Maternal Age**					
<20	20 (17.7)	75 (66.4)	18 (15.9)	113 (100)	
20–34	140 (10.3)	789 (58.2)	426 (31.4)	1355 (100)	
35–49	57 (11.6)	276 (56.3)	157 (32.0)	490 (100)	0.004
**Maternal Education**					
Primary and below	130 (19.4)	456 (68.2)	83 (12.4)	669 (100)	
Secondary and above	87 (6.7)	684 (53.1)	518 (40.2)	1289 (100)	0.002
**Ethnic Group**					
Hausa	96 (8.3)	656 (56.7)	404 (34.9)	1156 (100)	
Igbo	11 (19.3)	40 (70.2)	6 (10.5)	57 (100)	
Yoruba	47 (23.7)	133 (67.2)	18 (9.1)	198 (100)	
Others	63 (11.5)	311 (56.9)	173 (31.6)	547 (100)	<0.001
**Religion**					
Christianity	2 (0.7)	115 (38.0)	186 (61.4)	303 (100)	
Islam	215 (13.0)	1024 (61.9)	415 (25.1)	1654 (100)	<0.001
**Marital Status**					
Divorced	1 (4.5)	17 (77.3)	4 (18.2)	22 (100)	
Married/Cohabiting	173 (10.6)	931 (57.0)	530 (32.4)	1634 (100)	
Separated	2 (20)	5 (50)	3 (30)	10 (100)	
Never Married	38 (13.8)	175 (63.6)	62 (22.5)	275 (100)	
Widowed	3 (17.6)	12 (70.6)	2 (11.8)	17 (100)	0.016
**Socioeconomic Status**					
Low LS (0–3)	186 (16.1)	749 (64.8)	221 (19.1)	1156 (100)	
Middle LS (4–7)	29 (4.1)	362 (51.1)	318 (44.8)	709 (100)	
High LS (8–11)	2 (2.1)	29 (31.2)	62 (66.7)	93 (100)	<0.001
**Maternal Employment**					
Unemployed	89 (12.8)	425 (61.2)	180 (26.0)	694 (100)	
Employed	128 (10.1)	715 (56.6)	421 (33.3)	1264 (100)	0.002
**Paternal Education**					
Primary and below	97 (18.5)	352 (67.0)	76 (14.5)	525 (100)	
	217 (11.1)	1140 (58.2)	601 (30.7)	1958 (100)	
Secondary and above	76 (6.9)	579 (52.2)	454 (40.9)	1109 (100)	<0.001
**Paternal Employment**					
Unemployed	88 (14.4)	396 (64.6)	129 (21.0)	613 (100)	
Employed	129 (9.6)	744 (55.3)	472 (35.1)	1345 (100)	<0.001
**Birth Order**					
1^st^	66 (8.7)	397 (52.2)	297 (39.1)	760 (100)	
2^nd^	58 (11.3)	291 (56.5)	166 (32.2)	515 (100)	
3^rd^	84 (13.1)	420 (65.5)	137 (21.4)	641 (100.0)	<0.001
**Child Gender**					
Female	103 (10.8)	541 (56.6)	311 (32.6)	955 (100)	
Male	114 (11.4)	599 (59.7)	290 (28.9)	1003 (100)	0.216

LS = Living Standard.

Maternal age was significantly associated with care category (*p* = 0.004). Women under 20 years were least likely to receive complete care (16%) and most likely to fall in the no care group (18%). Older women (35–49) showed higher complete care uptake (32%). Maternal education strongly predicted care utilization (*p* = 0.002), with secondary education or above having substantially higher complete care uptake (40%) compared to those with primary or no education (12%). Ethnicity also showed significant differences (*p* < 0.001). Women from Hausa and Other ethnic groups had the highest rates of complete care (35% and 32%, respectively).

Religious affiliation was a strong predictor (*p* < 0.001). While 61% of Christian women received complete care, only 25% of Muslim women did. SES was significantly associated with care utilization (*p* < 0.001). Among women from high-SES households, 67% received complete care, compared to just 19% among those from low-SES households. Employed women were more likely to receive complete care (33%) than unemployed women (26%) (*p* = 0.002). Higher paternal education and employment status were significantly associated with receiving complete care (*p* < 0.001). Birth order showed a significant inverse relationship with complete care (*p* < 0.001), but child gender was not significantly associated with care category.

Table 4 presents the results of the multinomial logistic regression analysis examining predictors of receiving no care or partial care, with complete care serving as the reference category. The logistic regression models controlled for state of residence, maternal age, maternal education, ethnic group, religion, marital status, household socioeconomic status, maternal employment, paternal education, and child birth order. Variables included in the model were selected based on their statistical significance in bivariate analyses. Notably, significant geographic disparities were observed. Compared to women residing in Niger, those in Kano and Katsina had markedly higher odds of receiving no care (OR = 5.56, *p* < 0.001; OR = 13.73, *p* < 0.001). Similarly, women in these states were significantly more likely to receive partial care (OR = 2.19 and OR = 2.35, respectively; both *p* < 0.001). In contrast, women in Gombe had lower odds of partial care (OR = 0.51, *p* = 0.000), suggesting a higher likelihood of complete care in that state.

**Table 4.** Multinomial logistic regression of factors associated with No care and partial care compared to complete care.

	No Care		Partial Care	
	OR (95% CI)	*p*-Value	OR (95% CI)	*p*-Value
**State**				
Gombe	1.24 (0.48–3.20)	0.660	0.51 (0.33–0.81)	0.000
Kaduna	1.28 (0.51–3.21)	0.600	1.06 (0.71–1.60)	0.770
Kano	5.56 (2.43–12.75)	0.000	2.19 (1.40–3.42)	0.000
Katsina	13.73 (6.01–31.39)	0.000	2.35 (1.48–3.75)	0.000
Lagos	1.78 (0.48–6.64)	0.390	0.81 (0.54–1.22)	0.310
Niger	Reference			
**Maternal Age**				
<20	2.19 (0.91–5.22)	0.080	2.26 (1.18–4.33)	0.010
20–34	1.30 (0.82–2.09)	0.270	1.51 (1.12–2.03)	0.010
35–49	Reference			
**Maternal Education**				
Primary and below	2.95 (1.91–4.56)	0.000	1.93 (1.41–2.65)	0.000
Secondary and above	Reference			
**Ethnic Group**				
Hausa	0.65 (0.45–0.93)	0.022	0.91 (0.73–1.14)	0.400
Igbo	5.03 (1.79-.14.18)	0.002	3.73 (1.55–8.98)	0.003
Yoruba	7.17 (3.88–13.26)	0.000	4.13 (2.44–7.00)	0.000
Others	Reference			
**Religion**				
Christianity	0.30 (0.06–1.45)	0.130	0.99 (0.67–1.44)	0.950
Islam	Reference			
**Marital Status**				
Divorced	0.17 (0.01–3.43)	0.250	0.67 (0.09–4.71)	0.680
Married/Cohabiting	0.36 (0.05–2.73)	0.320	0.47 (0.10–2.36)	0.360
Separated	0.85 (0.04–16.90)	0.920	0.63 (0.06–6.35)	0.700
Single	0.21 (0.03–1.60)	0.130	0.36 (0.07–1.80)	0.210
Widowed	Reference			
**Socioeconomic Status**				
High	0.53 (0.12–2.41)	0.410	0.56 (0.33–0.93)	0.020
Middle	2.81 (1.72–4.58)	0.000	1.5 (1.16–1.94)	0.000
Low	Reference			
**Maternal Employment**				
Yes	1.18 (0.80–1.74)	0.390	1.16 (0.90–1.49)	0.250
No	Reference			
**Paternal Education**				
Primary and below	2.11 (1.33–3.35)	0.000	1.62 (1.17–2.26)	0.000
Secondary and above	Reference			
**Paternal Employment**				
Yes	–1.75)	0.860	1.17 (0.82–1.68)	0.380
No	Reference			
**Birth Order**				
1^st^	0.73 (0.44–1.20)	0.210	0.64 (0.46–0.88)	0.010
2^nd^	1.06 (0.64–1.75)	0.820	0.84 (0.60–1.15)	0.280
3^rd^	Reference			

OR = Odds Ratio; CI = Confidence Interval.

Maternal age was also associated with care utilization. Women aged < 20 years were more likely to receive partial care (OR = 2.26, *p* = 0.01) compared to those aged 35–49 years.

Education emerged as a strong predictor. Women with primary education or less had significantly higher odds of receiving no care (OR = 2.95, *p* < 0.001) and partial care (OR = 1.93, *p* < 0.001), compared to women with secondary education or higher. Ethnic identity significantly influenced care levels. Compared to women from other ethnic groups, Igbo and Yoruba women had significantly higher odds of receiving no care (OR = 5.03 and 7.17, respectively) and partial care (OR = 3.73 and 4.13, respectively), all *p* < 0.01. In contrast, Hausa women were significantly less likely to receive no care (OR = 0.65, *p* = 0.022), with no significant difference in partial care.

SES was a significant determinant. Women in the middle SES category had higher odds of both no care (OR = 2.81, *p* < 0.001) and partial care (OR = 1.50, *p* < 0.001). Conversely, high SES was protective against partial care (OR = 0.56, *p* = 0.02). Paternal education was also influential with women whose partners had only primary education were more likely to receive no care (OR = 2.11, *p* < 0.001) or partial care (OR = 1.62, *p* < 0.001). Although maternal and paternal employment were not significantly associated with care level, trends suggested better outcomes among employed women and partners. Finally, birth order was inversely associated with complete care. First-born children had significantly lower odds of receiving partial care (OR = 0.64, *p* = 0.01), while second-born children showed no significant differences. In contrast, child gender and religion were not significantly associated with differences in care category in the multivariate model.

## Model diagnostics and assumptions

Regression diagnostics confirmed that the model met key statistical assumptions. Multicollinearity assessment revealed no concerning relationships among predictor variables. All VIF values ranged from 1.04 to 1.45, well below the threshold of 10 that would indicate problematic multicollinearity (see supplement).

## Discussion

This study shows how patterns of care utilization along the maternal-child health continuum vary and significantly influence immunization outcomes. Our analysis revealed that women who received fewer than four ANC visits, delivered at home and with un-skilled birth attendant had markedly higher odds of having unvaccinated children. Overall, substantial gaps in the continuum of care were observed, with disparities driven by socioeconomic, demographic, and geographic factors.

These findings align with global and Nigerian literature, with studies from divers settings showing that access to maternal health services during pregnancy and childbirth are strongly associated with full immunization uptake [26–30]. In Nigeria, previous demographic surveys have consistently shown that ANC attendance and institutional delivery are strongly associated with higher rates of timely childhood immunization [31]. Other Nigerian studies also emphasize the mediating role of skilled birth attendance in linking mothers to postnatal services, including immunization [31,32].

Multiple mechanisms underpin these associations between maternal health services and child immunization uptakes. Services like ANC provide repeated opportunities for maternal education, confidence-building, immunization counseling, and scheduling of early childhood vaccinations. Facility delivery ensures immediate initiation of vaccines such as Bacillus Calmette – Guérin vaccine (BCG) and Oral Polio Vaccine (OPV), and skilled providers are more likely to adhere to immunization protocols and ensure newborn registration [33]. Conversely, home births and reliance on TBAs often result in missed contacts with formal systems, particularly in northern Nigeria, where civil registration and immunization card use remain low. This gap contributes to a persistent high rates of zero-dose children, a concern highlighted by both UNICEF [34] and the Immunization Agenda 2030 [34].

Our study further illustrates widespread differences in maternal and child health service uptake. Across the 6 states, only 31% of women received complete care, while 58% received partial care and 11% received none. These findings align with 2024 NDHS data, which show high ANC initiation but considerable attrition in the care continuum [16]. Partial care reflects fragmented service delivery, while the proportion receiving no care points to deeply entrenched structural and sociocultural barriers. This pattern is mirrored globally, with studies by Adedokun et al., Gryseels et al., Bhutta et al., and Ahmed et al. confirming that care drop-offs are prevalent in resource-limited settings and substantially contribute to avoidable maternal and neonatal mortality [33,35–37].

Our state-level analysis revealed pronounced geographic disparities. Women in Kano and Katsina had almost 6 and 14 times higher odds of receiving no care, respectively, compared to Niger state. In contrast, Lagos showed in this context better care outcomes, with 65% of women receiving complete care compared to only 12% in Kano and 11% in Katsina. These geographic differentials mirror the NDHS 2024 findings, which show facility delivery coverage as low as 19% in the Northwest compared to 83% in the Southwest [16]. Similarly, zero-dose prevalence remains highest in Katsina, Kano, and Sokoto, according to UNICEF [38].

These geographical disparities reflect a confluence of supply- and demand-side constraints. The Northern states faces chronic underinvestment in health infrastructure, persistent workforce shortages, and weak supply chains [39,40]. On the demand side, social barriers, such as low female education, religious conservatism, and patriarchal norms, impede women’s access to skilled care [41]. Opara et al. and Babalola et al. highlight how gendered and cultural decision-making and trust in TBAs obstruct the utilization of facility-based services [42,43]. In contrast, states like Lagos which are located in the southern part of Nigeria benefit from better infrastructure, higher literacy rates, and broader insurance penetration.

Addressing these disparities requires regionally adapted, equity-focused programming. As Kruk et al. and Bhutta et al. argue, reducing maternal-child health inequalities demands integrated investments in health systems, social protection, and local engagement [36,44]. Interventions such as mobile outreach, Community Health Influencers, Promoters, and Services deployment, and targeted social assistance must be scaled in poor-performing regions [36,44].

Interestingly, our study found that Hausa women were less likely to receive no care, which contrasts with earlier findings of lower service utilization among Northern ethnic groups [42,45]. This divergence may reflect recent maternal health interventions or improved outreach within Hausa communities and highlights the importance of localized, context-specific analyses. However, this finding should be interpreted cautiously, as the Hausa group had a substantially larger sample size (*n* = 1,156) compared to Yoruba (*n* = 198) and Igbo (*n* = 57), potentially resulting in more stable estimates for Hausa and less precision for the smaller groups. Further research with more balanced representation is needed to validate these results.

Our analysis also confirms the strong influence of individual and household characteristics. Multinomial regression revealed that women with only primary education were nearly three times more likely to receive no care and twice as likely to receive partial care compared to women with secondary education or higher. Similarly, women from low-SES households had three times higher odds of receiving no care, with only 19% of such households achieving complete care versus 67% among high-SES households. These findings align with other studies from Nigeria and other low and middle income countries, all of which confirm education and SES as powerful enablers of care-seeking behavior [42,46–48].

Our analysis also revealed critical age and parity effects. Adolescent mothers (<20 years) faced a heightened odd of receiving no or partial care. Moreover, care utilization declined with increasing birth order, with first-born children more likely to receive complete care (39%) than later-born children. These patterns suggest a combination of informational, experiential, and economic barriers. Adewuyi et al. and Wang and Mallick also report that younger and higher-parity mothers are particularly disadvantaged in care uptake [49,50].

Notably, paternal education emerged as a significant determinant of care utilization in this study. Women whose partners had only primary education were twice as likely to receive no care, highlighting the influence of male partners in maternal health decisions, particularly in patriarchal settings like Nigeria. This aligns with prior studies showing that educated men are more likely to support facility-based care and child immunization [49,51–53]. These findings underscore the need for male-inclusive RMNCH programming through community engagement, joint ANC counseling, and male-focused health education to improve maternal and child health outcomes.

A major strength of this study lies in its comprehensive approach to maternal and child health, examining service uptake across the full continuum of care, from antenatal visits to child immunization, rather than focusing on isolated outcomes. This integrated perspective captures the interdependence of care components and highlights systemic gaps, particularly in relation to zero-dose children. The reduced sample size may have limited statistical power and the precision of some estimates, particularly for subgroup analyses. However, the study’s explicit focus on social determinants provides important insights into how intersecting disadvantages shape maternal and child healthcare utilization.

Methodologically, the study employed a multi-stage probability sampling design, rigorous training and quality assurance procedures, and standardized digital data collection tools, ensuring data reliability and representativeness. The use of multinomial logistic regression provided robust insights into the predictors of no, partial, and complete care, making the findings highly relevant for both policy and program design. Importantly, the study aligns with Nigeria’s PHC revitalization agenda and global goals for universal health coverage, offering actionable evidence for equity-focused interventions.

Nonetheless, the study has limitations. Its cross-sectional design restricts causal inference and may not fully capture dynamic or seasonal variations in care-seeking behavior. Reliance on self-reported data introduces the possibility of recall and social desirability biases, particularly for sensitive indicators such as home delivery or immunization status. The absence of facility-level data and service quality metrics also limits the ability to interpret supply-side constraints, and residual confounding from unmeasured variables, such as transportation barriers or perceived quality of care, may persist despite statistical adjustments. Importantly, the selection of BHCPF-supported communities limits the generalizability of the findings to the broader Nigerian population. These communities may have received enhanced funding or support as stipulated under the BHCPF federal program, potentially leading to better-than-average outcomes in the four care indicators assessed.

## Conclusion

Our study highlights that women with fewer than four ANC visits, those delivering at home, and those attended by unskilled providers were significantly more likely to have zero-dose children, underscoring the interdependence of maternal health service uptake and child immunization outcomes. It also highlights substantial disparities in maternal and child healthcare utilization along the continuum of care in Nigeria, driven by geographic, socioeconomic, educational, and cultural factors. Our findings demonstrate a need for integrated, equity-focused interventions that strengthen early engagement with ANC, promote facility-based deliveries, and ensure postnatal linkage to immunization services. Targeted efforts are particularly needed in northern states and marginalized populations. Addressing these service gaps through community-based strategies, financial protection mechanisms, and multi-sectoral collaboration is essential to improving maternal and child health outcomes and achieving Nigeria’s universal health coverage and immunization equity goals.

## Supplementary Material

Supplement_27082025.docx

STROBE_checklist_cross_sectional.docx

## Data Availability

All data underlying reported findings have been made publicly available through figshare: 10.6084/m9.figshare.29571932
