# Supplementary material for: Determinants and gaps influencing zero-dose immunization and maternal Health service utilization in Nigeria: a cross-sectional household survey across six Nigerian states
Source: Glob Health Action. 2026 Apr 24;19(1):2660488. doi: 10.1080/16549716.2026.2660488 (PMC13112900; doi:10.1080/16549716.2026.2660488)
Supplement: Supplement_27082025.docx [file ZGHA_A_2660488_SM8856.docx]

**Supplement**: **Model Diagnostics and Assumptions**

The mean VIF was 1.26, substantially lower than the concerning threshold of 6. Correspondingly, tolerance values ranged from 0.69 to 0.96, all comfortably above the 0.1 threshold that would suggest multicollinearity issues (Table 5S). The highest VIF values were observed for maternal education (1.45) and socioeconomic status (1.39), followed by birth order and paternal education (both 1.36). However, these values indicate only minimal shared variance among predictors and do not compromise model validity.

Variables are ordered by VIF values (highest to lowest). VIF = Variance Inflation Factor; √VIF = Square root of VIF; Tolerance = 1 - R²; R² = Coefficient of determination when each variable is regressed on all other predictors. All VIF values are well below the problematic threshold of 10.0, indicating absence of multicollinearity concerns (O’brien et al. 2007)(23).

**Table 5:** Multicollinearity Diagnostics for Predictor Variables

| Variable | VIF | √VIF | Tolerance | R² |
| --- | --- | --- | --- | --- |
| Maternal education | 1.45 | 1.21 | 0.689 | 0.312 |
| Socioeconomic status | 1.39 | 1.18 | 0.718 | 0.282 |
| Birth order | 1.36 | 1.17 | 0.733 | 0.267 |
| Paternal education | 1.36 | 1.16 | 0.738 | 0.263 |
| Maternal age | 1.26 | 1.12 | 0.792 | 0.208 |
| Religion | 1.21 | 1.10 | 0.828 | 0.172 |
| State | 1.12 | 1.06 | 0.891 | 0.110 |
| Maternal employment | 1.11 | 1.05 | 0.902 | 0.098 |
| Paternal employment | 1.04 | 1.02 | 0.959 | 0.041 |
